# Chloroplast Genomes Evolution and Phylogenetic Relationships of *Caragana* species

**DOI:** 10.3390/ijms25126786

**Published:** 2024-06-20

**Authors:** Xingyong Cui, Kangjia Liu, Enze Li, Zhixiang Zhang, Wenpan Dong

**Affiliations:** School of Ecology and Nature Conservation, Beijing Forestry University, Beijing 100083, China; cuixy@bjfu.edu.cn (X.C.); liukangjia@bjfu.edu.cn (K.L.); lienze@bjfu.edu.cn (E.L.)

**Keywords:** inverted repeat loss clade, SSRs and dispersed repeats, codon usage, substitution rate, phylogeny

## Abstract

*Caragana* sensu lato (*s.l.*) includes approximately 100 species that are mainly distributed in arid and semi-arid regions. *Caragana* species are ecologically valuable for their roles in windbreaking and sand fixation. However, the taxonomy and phylogenetic relationships of the genus *Caragana* are still unclear. In this study, we sequenced and assembled the chloroplast genomes of representative species of *Caragana* and reconstructed robust phylogenetic relationships at the section level. The *Caragana* chloroplast genome has lost the inverted repeat region and wascategorized in the inverted repeat loss clade (IRLC). The chloroplast genomes of the eight species ranged from 128,458 bp to 135,401 bp and contained 110 unique genes. All the *Caragana* chloroplast genomes have a highly conserved structure and gene order. The number of long repeats and simple sequence repeats (SSRs) showed significant variation among the eight species, indicating heterogeneous evolution in *Caragana*. Selective pressure analysis of the genes revealed that most of the protein-coding genes evolved under purifying selection. The phylogenetic analyses indicated that each section forms a clade, except the section *Spinosae*, which was divided into two clades. This study elucidated the evolution of the chloroplast genome within the widely distributed genus *Caragana.* The detailed information obtained from this study can serve as a valuable resource for understanding the molecular dynamics and phylogenetic relationships within *Caragana*.

## 1. Introduction

*Caragana* Fabr. belongs to the Fabaceae and includes approximately 100 species [1]. Its native distribution ranges from Eastern Europe to temperate Asia, including Central Asia, the Qinghai–Tibet Plateau, and East Asia [2]. The genus prefers arid and cold habitats, being mainly distributed in arid and semi-arid regions [3], where it is often the dominant plant in the community [4].

**Figure 1:**
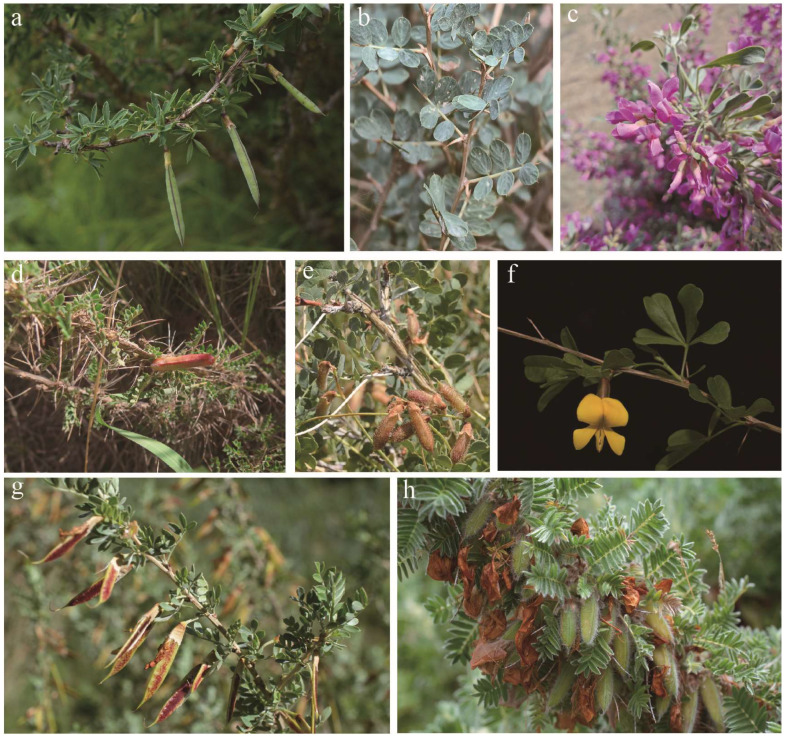
The morphology of the species sequenced in this study. (**a**) *Caragana erinacea*; (**b**) *C. acanthophylla*; (**c**) *C. halodendron*; (**d**) *C. roborovskyi*; (**e**) *Calophaca soongorica*; (**f**) *C. rosea*; (**g**) *C. korshinskii*; (**h**) *C. jubata*.

The taxonomy and phylogenetic relationships of the genus *Caragana* sensu lato (*s.l.*) are still unclear. Zhao [5] divided *Caragana* sensu stricto (*s.s.*) into five sections according to morphological characteristics (namely, *Caragana*, *Spinosae*, *Longispinae*, *Jubatae*, and *Frutescentes*). Recently, Duan et al. [6], using the nuclear ITS and plastid *matK*, *psbA*-*trnH*, and *trnL*-*F* markers, supported that the two genera of *Calophaca* and *Halimodendron* belong to *Caragana* and should be treated as section *Calophaca* and section *Halimodendron*. *Calophaca* Fisch. includes about eight species with a native range from European Russia to Xinjiang and Pakistan [7]. *Halimodendron* Fisch. ex DC. includes only one species, which is distributed in dry sand and saline soil in the Caucasus, Western Siberia, Central Asia, and the Tianshan Mountains. Both *Caragana s.s.* and *Calophaca* are shrubs. *Caragana s.s.* has paripinnate leaves; axillary flowers that are usually solitary but sometimes present in groups of two–five in a fascicle; and cylindrical or compressed legumes. *Calophaca* has imparipinnate leaves and four-flowered (or more) raceme (Figure 1). *Halimodendron* has paripinnate leaves; racemes with four or more flowers; inflated legumes; and thick valves [8].

The phylogenetic relationships among the sections of *Caragana* sensu lato (*s.l.*) were poorly supported and could not be resolved using the *rbcL*, *trnS*–*trnG*, and *ITS* markers [4]. Moreover, the topology of the section was also inconsistent between different studies. For example, section *Caragana* or *Frutescentes* was supported as the basal group of *Caragana* based on the results of Zhang et al. [9] and Duan et al. [6], respectively. Previous studies suffered from poor phylogenetic resolution due to the limited availability of DNA sequence data (*rbcL*, *matK*, *trnS–trnG*, *trnL–F*, *psbA–trnH*, and ITS) and the low genetic variability in the selected molecular markers [6,10,11], possibly attributed to the recent origin and rapid diversification of this genera. Overall, previous phylogenetic trees showed problems such as low bootstrap values and low robustness due to only a few molecular markers with the limitation of sequencing technology. Consequently, expanding the sampling of genetic information, such as by using complete chloroplast genomes, could provide a more robust phylogenetic tree and could improve investigations of the evolutionary history of *Caragana* at the section level. Furthermore, a well-supported phylogenetic framework will enable a better estimate of the divergence time of these genera.

Chloroplast genome sequences have been widely used to investigate phylogenetic relationships among closely related species due to their higher mutation rates, maternal inheritance, and lack of recombination [12,13,14]. The advancement of sequencing technologies has facilitated cost-effective, easy-to-obtain, complete chloroplast genome sequences, enabling the transition from gene-based phylogenetics to genome-based phylogenomics [13,15] and facilitating the investigation of evolutionary phenomena in plant species with greater efficiency. Recently, the chloroplast genomes of several *Caragana s.s.* species have been sequenced and annotated, focusing on chloroplast genome evolution [16,17,18].

In order to understand the evolutionary history and the dynamic evolution of *Caragana*, we sequenced the chloroplast genomes of representative species of *Caragana*. In this study, we specifically aimed to (1) deepen our understanding of *Caragana* evolution using chloroplast genomes and (2) reconstruct robust phylogenetic relationships in *Caragana* at the section level.

## 2. Results

### 2.1. Characteristics of Caragana Chloroplast Genomes

**Figure 2:**
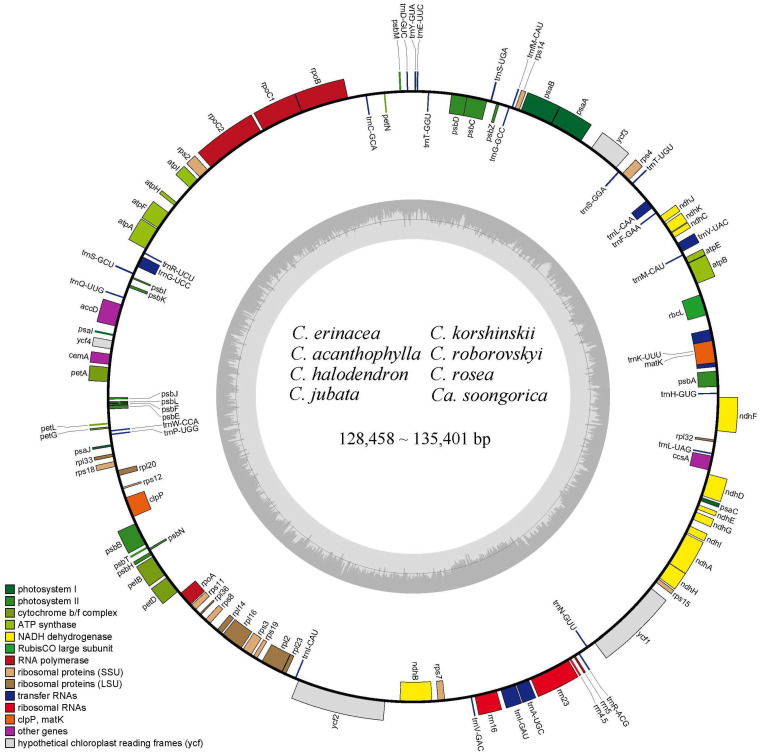
Gene map of the chloroplast genomes of *Caragana* species. Genes outside the ring are transcribed in a counterclockwise direction, whereas those inside the ring are transcribed in a clockwise direction. The GC content of the chloroplast genome is shown in the dark gray region, and the AT content is shown in the light gray region. *Calophaca soongorica* has the abbreviation of *Ca. soongorica*.

The lengths of newly sequenced chloroplast genomes of *Caragana* varied from 12.8458 kb (*C. jubata*) to 13.5401 kb (*C. rosea*) (Table 1). All *Caragana* chloroplast genomes had lost the IR region and did not have the typical quadripartite structure found in most land plant chloroplast genomes (Figure 2). The GC content of the eight *Caragana* chloroplast genomes ranged from 34.3% (*C. korshinskii*) to 34.8% (*C. rosea*).

We annotated 110 unique genes, including 76 protein-coding genes, 30 tRNA genes, and four rRNA genes in *Caragana* chloroplast genomes (Figure 2). The genes could be divided into three categories. We identified 57 genes associated with self-replication, encompassing transfer RNA, ribosomal RNA, and three subunits—large, small, and DNA-dependent RNA polymerase—encoding chloroplast RNA polymerase. Additionally, 44 genes were linked to photosynthesis. The remaining genes were classified as either other genes or unknown genes. A total of 17 genes had introns; *ycf3* had two introns, and the remaining 16 genes (*atpF*, *clpP*, *ndhA*, *ndhB*, *petB*, *petD*, *rpl2*, *rpl16*, *rpoC1*, *rps12*, *trnA*-*UGC*, *trnG*-*UCC*, *trnI*-*GAU*, *trnK*-*UUU*, *trnL*-*CAA*, and *trnV*-*UAC*) had only one intron.

### 2.2. Analyses of Repeat Sequences

**Figure 3:**
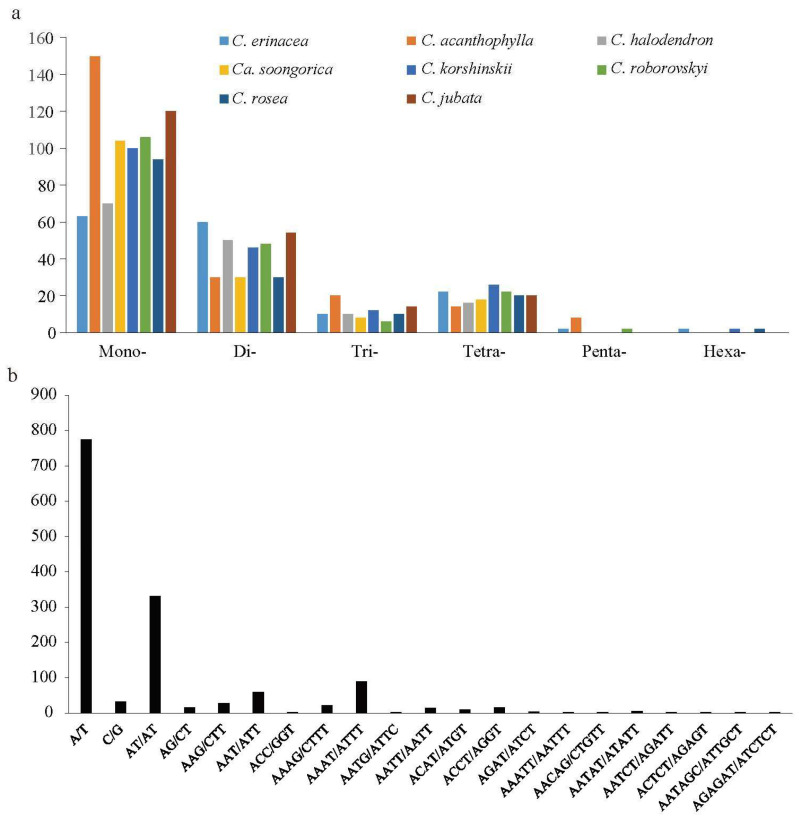
Number and type of simple sequence repeats (SSRs) of eight *Caragana* species in the chloroplast genome. (**a**) Number of six types of SSRs. (**b**) Number of base types of SSRs.

In the eight *Caragana* chloroplast genomes, we detected 1421 simple sequence repeats (SSRs), with the number of SSRs in each genome ranging from 156 to 222 (Figure 3). Six types of SSR were identified. Among them, mononucleotide repeats were the most frequent in all eight species, with proportions ranging from 39.62% (*C. erinacea*) to 67.57% (*C. acanthophylla*) (Figure 3a). Dinucleotide repeats were the second most frequent SSR type, with proportions ranging from 13.51% (*C. acanthophylla*) to 37.74% (*C. erinacea*). *Caragana acanthophylla* had the most trinucleotide repeats, followed by *C. jubata* with 14 trinucleotide repeats. The number of tetranucleotide repeats ranged from 14 (*C. acanthophylla*) to 26 (*C. korshinskii*). Some *Caragana* species did not have trinucleotides or hexanucleotides. The SSRs had a significant bias toward A/T bases (Figure 3b), with most mononucleotide repeats being A/T (54.54%) and most dinucleotide repeats being AT/TA (23.36%).

**Figure 4:**
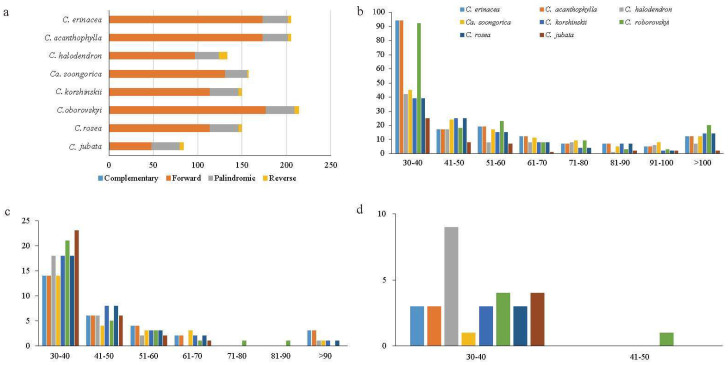
Number and type of dispersed repeats of eight *Caragana* species in the chloroplast genome. (**a**) Number and type of long repeats in dispersed repeats. (**b**) Length and frequency of forward long repeats. (**c**) Length and frequency of palindromic long repeats. (**d**) Length and frequency of reverse long repeats.

We detected 1296 long repeats, consisting of forward repeats (F), palindromic repeats (P), reverse repeats (R), and complementary (C) repeats with 30–472 bp in the eight chloroplast genomes (Figure 4a). The repeat number and length varied from one to another. *Caragana roborovskyi* had the greatest number of repeats (314) followed by *C. acanthophylla* and *C. erinacea* (205), and *C. jubata* had the lowest (84). The number of long repeats significantly differed among the four long repeat types. Forward repeats were most common (1025), followed by palindromic repeats (238). Complementary repeats were only identified in *C. jubata* and *C. roborovskyi*. The analysis of forward, palindromic, and reverse repeat lengths revealed that the most common type was long repeats (30–40 bp; Figure 4b–d), followed by repeats with lengths of 41–50 bp and 51–60 bp.

### 2.3. Genomic Sequence Divergence

**Figure 5:**
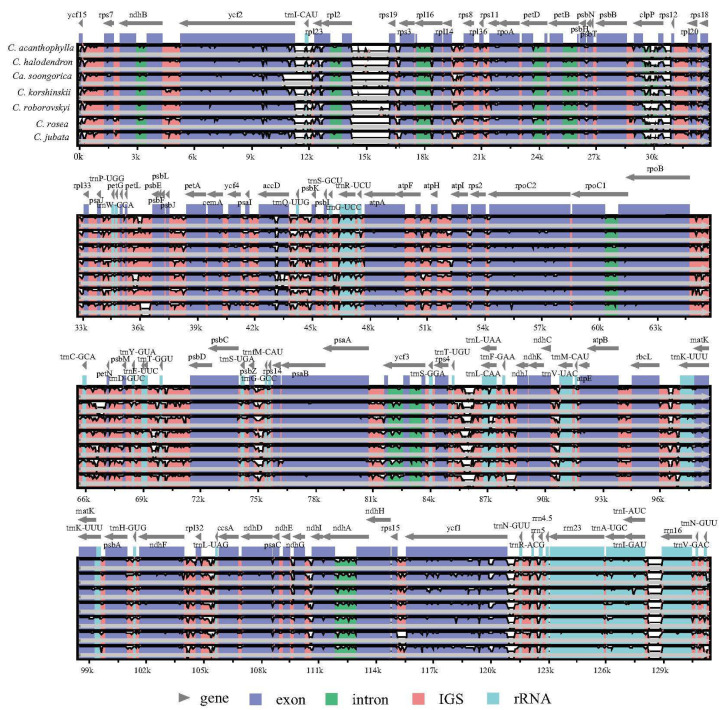
Sequence similarity plots of eight chloroplast genomes of *Caragana,* which were constructed using mVISTA (*C. erinacea* was used as the reference sequence). The proportion of sequence identity is displayed on the y-axis and ranges from 50% to 100%. The x-axis indicates the co-ordinates of the chloroplast genomes. Genomic regions are color-coded as protein-coding (exon), intron, intergenic region space (IGS), and rRNA. Genes are indicated by gray arrows.

The multiple sequence alignments determined with mVISTA revealed no evidence of genomic rearrangements or large inversions across the *Caragana* chloroplast genomes, as illustrated in Figure 5. These chloroplast genomes exhibited a high degree of conservation, both in terms of gene order and sequence identity. Notably, the coding regions and inverted repeat (IR) regions demonstrated a higher level of conservation compared to the non-coding regions and single-copy regions. The analysis further revealed that the intergenic spacer regions exhibiting the most variation were *trnI*-*rpl23*, *rpl2*-*rps19*, *clpP*-*rps12*, *trnC*-*petN*, *trnT*-*trnL*, and *trnN*-*trnR*. Additionally, the coding genes *accD*, *rpoC2*, and *ycf1* demonstrated high levels of variation among the coding regions of the chloroplast genomes.

### 2.4. Codon Usage Pattern and Molecular Evolution

We analyzed the codon usage of the protein-coding genes in the chloroplast genomes of the eight *Caragana* species (Table S3). The number of codons ranged from 23,335 (*Calophaca soongorica*) to 23,750 (*C. halodendron*). We identified 64 codons encoding 20 amino acids, including three termination codons. Leucine (Leu) showed the highest abundance among the amino acids, accounting for 24,164 occurrences (12.89%) across all eight species. This was followed by isoleucine (Ile), with a frequency of 15,339 (8.18%). In contrast, methionine (Met) was represented by the fewest number of codons, totaling 5017 occurrences.

**Figure 6:**
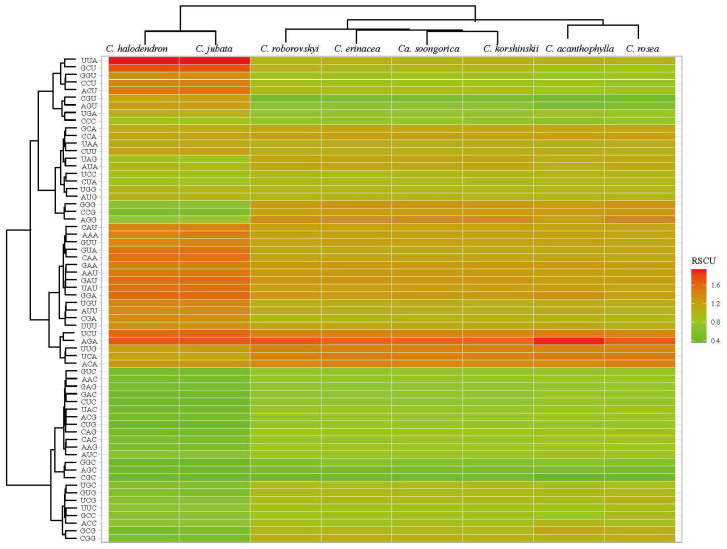
The relative synonymous codon usage (RSCU), calculated for eight species using CodonW.

The results of relative synonymous codon usage (RSCU; Figure 6) indicate that 24 codons were used more frequently with RSCU > 1. Notably, 23 of these 24 codons possessed A/U at the third nucleotide positions except UUG. Conversely, most of the codons ending with G/C exhibited RSCU values lower than 1, suggesting less prevalent usage in the eight chloroplast genomes. The bias towards A/U at the third position of the codons was further evidenced by the A/U contents of the codons, with a mean value of 58.79% at the third codon position. Two codons, ATU for methionine (Met) and UGG for tryptophan (Trp), displayed RSCU values of 1.00, indicating no codon bias.

### 2.5. Molecular Evolution of the Caragana Chloroplast Genome

**Figure 7:**
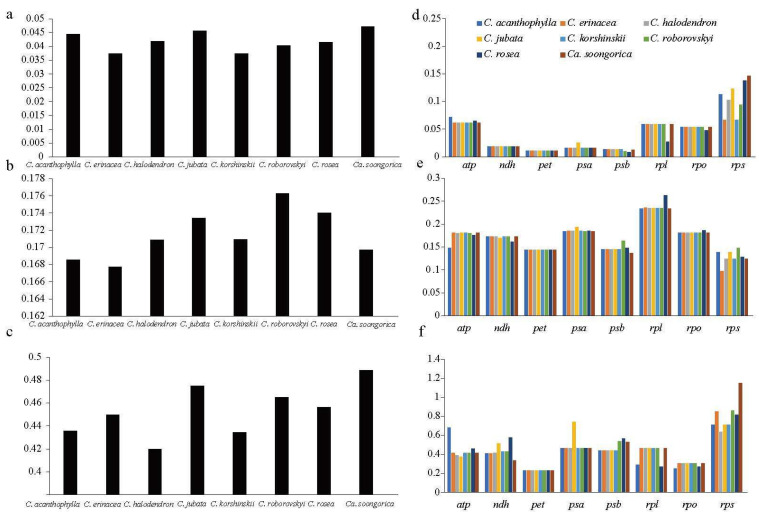
The non-synonymous substitution ratio (*dN*), synonymous substitution ratio (*dS*), and *dN/dS* ratio (ω), computed for eight species and gene groups using PAML. (**a**–**c**) *dN*, *dS*, and ω values of eight species. (**d**–**f**) *dN*, *dS*, and ω values of gene groups with eight species.

We assessed the evolutionary rate disparities among *Caragana* species according to computed *dN*, *dS*, and ω values with 74 protein-coding genes, gene groups, and the combinations of all 74 protein-coding genes for each *Caragana* species (Figure 7 and Table S4). The *t*-test for *dN*, *dS*, and ω values revealed noteworthy variations for each gene, denoting diverse molecular evolution rates across genes.

The highest *dN* value was linked to *accD* (0.37), and the mean *dN* value was 0.052. Notably, three genes (*petD*, *petN*, and *psaC*) exhibited non-synonymous substitution sites with *dN* = 0. The mean *dS* value was 0.18, and *accD* displayed the highest *dS* value (0.78), followed by *psbL* (0.65) and *clpP* (0.43). Most ω values were <1, indicating that most genes were predominantly under purifying selection. However, positive selection was observed in 13 genes (*atpF*, *atpI*, *cemA*, *matK*, *ndhB*, *psaI*, *rpl20*, *rps14*, *rps7*, *rps8*, *ycf1*, *ycf2*, and *ycf4*), with at least one species-pair exhibiting ω > 1.

Among the different gene groups (Figure 7d), the *rps* group exhibited the highest *dN* value (0.106), followed by *atp* (0.063) and *rpl* (0.055), while the photosynthetic genes (*psa* and *psb*), *ndh* and *pet* demonstrated the lowest *dN* values. There was less variation in *dS* among the different gene groups (Figure 7e). The ω value was <0.5 for all gene groups except the *rps* group, indicating purifying selection (Figure 7f). The ω value was >1 in the *rps* group in *Calophaca soongorica*, indicating positive selection within this group.

At the species level (Figure 7a), *Calophaca soongorica* showed the highest *dN* value (0.047), followed by 0.046 in *C. jubata* and 0.044 in *C. acanthophylla*. *Caragana roborovskyi* had the highest *dS* value (0.176) (Figure 7b). Both *dS* and *dN* values demonstrated less variability between the eight species. All ω values were <0.5 (ranging from 0.420 to 0.489), indicating a much lower frequency of non-synonymous substitutions compared to synonymous substitutions and that the species were under purifying selection (Figure 7c).

### 2.6. Phylogenetic Analysis

The genera *Caragana* included seven sections: *Caragana*, *Calophaca, Halimodendron, Spinosae*, *Longispinae*, *Jubatae*, and *Frutescentes*. A total of 26 *Caragana* (including genus *Halimodendron* and *Calophaca*) chloroplast genomes were used for phylogenetic investigation to elucidate the phylogenetic relationships of *Caragana* at the section level. The species *Astragalus tumbatsica*, *Astragalus mongholicus*, *Hedysarum taipeicum*, and *Hedysarum petrovii* were used as outgroups in the analysis.

**Figure 8:**
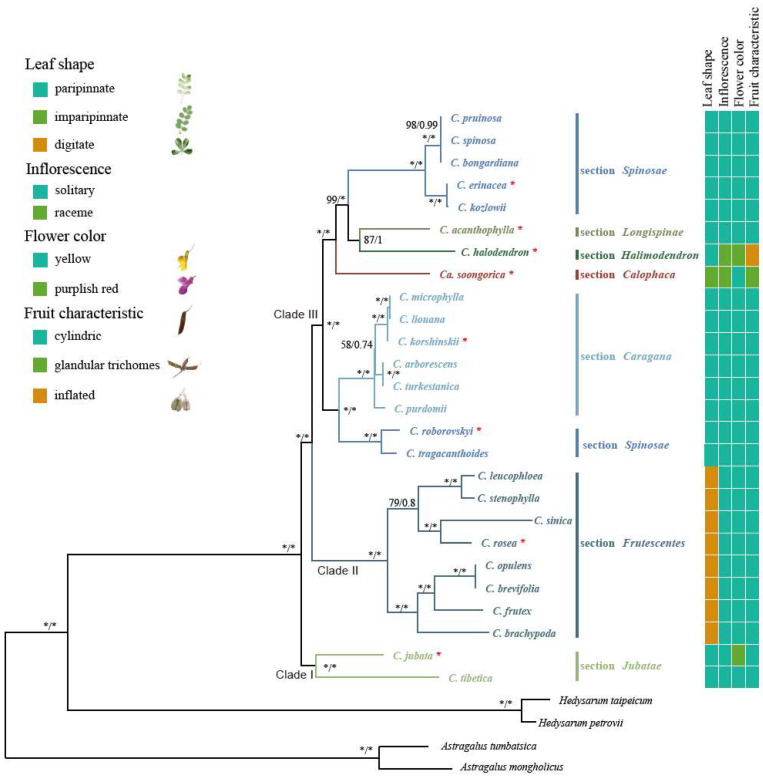
Phylogenetic tree, constructed using the maximum likelihood (ML) and Bayesian inference (BI) methods with chloroplast genomes. The number on the line represents the ML bootstrap support (BS) value and Bayesian posterior probability (PP) value for each node (in both, “*” in black font represents the BS 100 or PP 1.0). “*” in red font represents the newly sequenced chloroplast genomes. Important taxonomic characteristics are shown on the right side of the tree.

Both maximum likelihood (ML) and Bayesian inference (BI) approaches yielded consistent tree structures (Figure 8), with robust support for the monophyly of the genus *Caragana*, which was evident in both analyses (bootstrap value (BS) = 100%; posterior probability (PP) = 1.0). The *Caragana* phylogenetic trees were strongly supported, indicating that the relationships among the *Caragana* species were well resolved based on the whole chloroplast genome sequences. These species of *Caragana* formed three clades with high support. Clade I, consisting of the section *Jubatae*, was the first group of this genera to diverge. The section *Frutescentes* formed Clade II and was sister to the rest of *Caragana.* The section *Frutescentes* further diverged into two groups. Clade III included two main groups; section *Caragana* was sister to section *Spinosae* and formed a group. The four sections of *Calophaca*, *Halimodendron*, *Longispinae*, and *Spinosae* formed a clade with high support (BB = 100%; PP = 1.0), and section *Calophaca* was sister to the other three sections. The section *Spinosae* did not form a clade and was divided into two groups.

## 3. Discussion

### 3.1. Structure and Comparative Analysis of Caragana Chloroplast Genomes

In this study, we sequenced eight species (Figure 1) of *Caragana*, each from a different section, and we compared the chloroplast genome variations and mutations. The chloroplast genome length ranged from 128,458 bp to 135,401 bp, which is significantly shorter than other angiosperms [12,13,19]. The shorter genome lengths were due to the loss of the inverted repeat (IR) region (Figure 2). *Caragana*, as a member of the tribe Caraganeae, is categorized within the IR-lacking clade (IRLC) in the subfamily Papilionoideae of Fabaceae. The loss of the chloroplast genome IR region in IRLC taxa, which is recognized as a robust phylogenetic indicator within the clade, has been consistently validated in prior research [20,21,22].

The loss of IR regions likely leads to mechanisms such as increased mutation rates, facilitated genome rearrangements, and altered selection pressures and intron structure, significantly accelerating the rate of evolution of the chloroplast genome [23]. Numerous studies have shown gene and intron loss, such as the loss of introns from the *rps12* and *clpP* genes in chickpea (*Cicer arietinum*) [24], loss of the *accD* gene in *Trifolium* [25,26], and loss of *rpl22*, *rps16*, and one intron of *clpP* in *Vicia* [27]. In this study, the *rpl22* and *infA* genes in the *Caragana* chloroplast genomes were lost (Figure 2). The *infA* gene is a notably labile chloroplast gene, and there are multiple independent cases of its loss or transfer to the nucleus in flowering plants [28]. The gene *rpl22*, encoding ribosomal protein *CL22*, also has been lost from the chloroplast genomes and translocated to the nucleus [29].

The significant difference in chloroplast genome size between *Caragana* and other angiosperms indicates that IRLC chloroplast genomes may have undergone different evolutionary processes. Compared to other angiosperm species, the GC content in the *Caragana* chloroplast genome is lower due to the IR region generally displaying higher GC content compared to other regions of the chloroplast genome. This disparity is primarily attributed to the presence of four rRNA genes with high GC content (50–56.4%) in the IRs. Similar to GC content, codon usage biases also showed differences from other species, including both IR regions, indicating significant molecular evolutionary processes. Notably, *Caragana* chloroplast genomes exhibited similar GC content and codon usage bias to other IRLC species [30].

Repeat sequences play a crucial role in genome rearrangements and serve as significant markers in evolutionary studies [31]. We identified four types of long repeats and SSRs. Forward repeats were the most frequent in the *Caragana* chloroplast genomes, and complementary repeats were the lowest (Figure 4). There are minor variations in the numbers and types of repeats among closely related species (Figure 5). Nonetheless, the number of repeats displayed noteworthy variability across the eight *Caragana* species, indicating heterogeneous evolution in the *Caragana* chloroplast genomes. SSRs exhibit high levels of polymorphism at the species level. They have been extensively studied in the field of population genetics, and variation has been documented [32,33]. In the current study, mononucleotide repeats were highly abundant, predominantly comprising A/T repeats rather than G/C repeats (Figure 3). This strong bias toward A/T in SSR loci was also noted in other legumes, such as *Albizia julibrissin* [34] and *Desmodium stryacifolium* [35].

### 3.2. Phylogenetic Relationships among the Sections of Caragana

We reconstructed a robust phylogenetic tree with high bootstrap values using chloroplast genomes. The findings do not support the results of Zhao [5], who classified *Caragana* into three subgenera. The results of the current study support the monophyly of *Caragana* and classify *Calophaca* and *Halimodendron* as sections [6]. Previous studies have shown that the monophyly of sections *Longispinae*, *Spinosae*, *Frutescentes*, and *Jubatae* is difficult to recover based on the small number of molecular markers [11], but we recovered the monophyly of sections *Frutescentes* and *Jubatae*. Our results strongly suggest that *Caragana* be split into two clades with seven sections. Section *Jubata* was found to be a sister to the most recent common ancestor of the other six sections and was thus identified as the basal clade. The next to be differentiated was section *Frutescentes*. The sister relationship of section *Longispinae* and *Halimodendron* was not strong (bootstrap value = 87). The monophyly of section *Spinosae* was not recovered, and future studies with nuclear genetic data representing biparental inheritance and more complete species sampling will be required [36], as well as further study of the cause of conflict by comparing the phylogenetic relationships of nuclear genes and plastids.

Our phylogenetic results are consistent with the existing morphological taxonomic consensus [5]. Section *Calophaca* has some unique traits, such as imparipinnate leaves and racemes with glandular trichomes, and we speculated that these traits are derived characteristics. Section *Halimodendron* has racemes, pale purple or purplish red corollas, inflated legumes, and thick valves. It has a strong tolerance to drought and salinity, and its habitats vary greatly from those of other species in the genus *Caragana*. We propose that section *Calophaca* likely experienced a unique adaptive evolutionary history. The leaf morphology of *Caragana* is diverse and is mainly divided into paripinnate (4–20-foliolate) and digitate (4-foliolate). All members of section *Frutescentes* have digitated leaves. Section *Frutescentes* also has the highest species richness, covering 36% of *Caragana*, which is likely related to trait innovation. Section *Caragana* has caducous rachis, pinnate leaves, and 4–10 pairs of leaflets. Section *Jubata* has persistent rachis, pinnate leaves, and three–seven pairs of leaflets and is mostly distributed in high-elevation regions. The leaflets of section *Spinosae* are pinnate on the rachis of long branches, digitate or pinnate connected on the rachis of short branches, and are often found in two–three pairs. Section *Longispinae* has three–nine leaflet pairs and generally either long branches with persistent rachis or short branches with caducous rachis.

## 4. Materials and Methods

### 4.1. Plant Material, DNA Extraction, and Sequencing

According to the taxonomic system of *Caragana,* six *Caragana s.s.* species, one *Halimodendron* species (*Halimodendron halodendron*), and one *Calophaca* species (*Calophaca soongorica*) were selected for this study (Figure 1), representing all the eight sections of *Caragana.* Fresh leaves were collected from various regions in China, including Xinjiang, Xizang, Gansu, Sichuan, Hebei, and Beijing. The fresh leaves were dried with silica gel. Details on the material are provided in Table S1. The plant materials were identified by Prof. Zhixiang Zhang (Beijing Forestry University). Voucher specimens were deposited at the museum of Beijing Forestry University (BJFC) under voucher specimen numbers BJFC00024602, BJFC00120870, BJFC00120894, ZJK05210301, FS05270301, and SC060501.

Genomic DNA was extracted following the methods of Li et al. [37]. The quality and quantity of extracted DNA were evaluated with a Qubit 3.0 Fluorometer (Thermo Fisher Scientific, Waltham, MA, USA). Then, 300 ng of DNA for each sample was randomly sheared into ~350-bp fragments using an Ultrasound Covaris instrument. The paired-end sequencing library was generated and sequenced on the DNBSEQ-T7 platform to generate 150 bp paired-end reads. For each sample, approximately 5 GB of raw data was obtained.

### 4.2. Chloroplast Genome Assembly and Annotation

Trimmomatic 0.36 [38] was used to check the sequencing quality of the raw data, such as adapter-contaminated reads and low-quality bases. The chloroplast genomes were de novo assembled with the GetOrganelle v1.6.2 [39] with parameter k-mer lengths of 95. If GetOrganelle was unable to assemble a complete chloroplast genome, the method of Dong et al. [13,40] was used to complete the assembly. Plastid Genome Annotator (PGA) Version 3 [41] was used to annotate the newly sequenced chloroplast genomes. Geneious Prime was used to check and revise the annotation errors manually. The online software Chloroplast (https://irscope.shinyapps.io/Chloroplot/, accessed on 21 May 2024) was used to draw the physical maps.

### 4.3. Analysis of Repeat Sequences and SSRs

We used MISA (http://pgrc.ipk-gatersleben.de/misa/, accessed on 21 May 2024) to identify the simple sequence repeats (SSRs) within the eight *Caragana* species [42]. Six types of SSRs (mononucleotide, dinucleotide, trinucleotide, tetranucleotide, pentanucleotide, and hexanucleotide SSRs) were analyzed, and the repeat thresholds were set to 10, 5, 4, 3, 3, and 3, respectively. Moreover, we used REPuter (https://bibiserv.cebitec.uni-bielefeld.de/reputer/, accessed on 23 May 2024) to identify the four types of dispersed repeats (forward, palindromic, reverse, and complementary repeats). The parameters were set as follows: a repeat identity of more than 90%, a minimum repeat size of 30 bp, and a hamming distance of 3.

### 4.4. Comparative Genomic Analyses and Molecular Evolution Analysis

The *Caragana* chloroplast genome structure variation was examined using mVISTA (https://genome.lbl.gov/vista/mvista/submit.shtml, accessed on 23 May 2024) [43] with the shuffle-LAGAN model. The chloroplast genome of *Caragana erinacea* was used as the reference sequence.

We calculated relative synonymous codon usage (RSCU), which is the ratio of the observed frequency of a specific codon to the expected frequency, with CodonW v1.4.2. The R package *Pvclust* was used to assess the uncertainty in hierarchical cluster analysis [44]. The RSCU values were then plotted as a heatmap using TBtools 1.116 [45].

We computed the non-synonymous substitution ratio (*dN*), synonymous substitution ratio (*dS*), and *dN/dS* ratio (ω) to investigate the molecular evolution within the *Caragana* chloroplast genomes. This evaluation facilitates the examination of evolutionary rates and the impact of natural selection on molecular evolution. *Hedysarum taipeicum* served as the reference species in these analyses.

We extracted all protein-coding genes with PhyloSuite v1.2.3 according to genome annotation [46] and aligned with MAFFT v7.471. The *dN*, *dS*, and ω values were determined utilizing the YN100 method in PAML v4.10.3 [47]. These calculations were conducted for gene clusters sharing identical functions [48], and at the species level, they were combined with all coding genes for each species.

### 4.5. Phylogenetic Analysis

The phylogenetic relationships of *Caragana* were reconstructed using the chloroplast genome sequences. In addition to the chloroplast genomes sequenced in this study, complete *Caragana* chloroplast genomes (publicly available in NCBI) were used (Table S2). Four species from two genera were designed as outgroups according to a previous phylogenetic study [49]. We aligned the whole chloroplast genome sequences with MAFFT [50] with default parameters and detected the unreliable alignment regions with trimAl v1.4.1 [51].

We performed the phylogenetic analyses using two methods: maximum likelihood (ML) and Bayesian inference (BI). ModelFinder v1.5.4 was used to determine the best substitution model [52]. The ML tree was reconstructed using IQ-TREE v2.0.3 [53] with 1000 bootstrap replicates. The BI tree was inferred using MrBayes 3.2 [54]. Four MCMC simulations were run simultaneously and sampled every 1000 generations for a total of two million generations. The first 25% of each tree was discarded as burn-in. We used Tracer v1.7 [55] to analyze the effective size of each parameter and the convergence to a stationary distribution, and we estimated posterior probabilities using a 50% majority-rule consensus tree.

## Figures and Tables

**Table 1:** Features of the chloroplast genomes of eight *Caragana* species.

Species	Length (bp)	GC (%)	Number of Genes			
			Total	Coding Genes	tRNA	rRNA
*Caragana erinacea*	131,345	34.6	110	76	30	4
*Caragana acanthophyllaa*	130,523	34.5	110	76	30	4
*Caragana halodendron*	129,144	34.4	110	76	30	4
*Calophaca soongorica*	128,813	34.7	110	76	30	4
*Caragana korshinskii*	130,052	34.3	110	76	30	4
*Caragana roborovskyi*	130,392	34.4	110	76	30	4
*Caragana rosea*	135,401	34.8	110	76	30	4
*Caragana jubata*	128,458	34.4	110	76	30	4

## Data Availability

The chloroplast genome of *Caragana* is deposited in the GenBank database under the following accession number: PP848196, PP897764-PP897770.

## Supplementary Materials

The following supporting information can be downloaded at: https://www.mdpi.com/article/10.3390/ijms25126786/s1.

## References

[1] Roskov Y., Bisby F., Zarucchi J., Schrire B., White R.J.R. (2005). ILDIS World Database of Legumes: Draft Checklist.

[2] Lewis G.P., Schrire B., Mackinder B., Lock M. (2005). Legumes of the World.

[3] Du L.-T., Ma L.-L., Pan H.-Z., Qiao C.-L., Meng C., Wu H.-Y., Tian J., Yuan H.-Y. (2022). Carbon–water coupling and its relationship with environmental and biological factors in a planted Caragana liouana shrub community in desert steppe, northwest China. J. Plant Ecol..

[4] Zhang M.L., Xiang X.G., Xue J.J., Sanderson S.C., Fritsch P.W. (2016). Himalayan uplift shaped biomes in Miocene temperate Asia: Evidence from leguminous Caragana. Sci. Rep..

[5] Zhao Y. (2009). Classification and Its Floristic Geography of Caragana Fabr. in the World.

[6] Duan L., Yang X., Liu P.L., Johnson G., Wen J., Chang Z.Y. (2016). A molecular phylogeny of Caraganeae (Leguminosae, Papilionoideae) reveals insights into new generic and infrageneric delimitations. PhytoKeys.

[7] Zhang M.L., Wen Z.B., Fritsch P.W., Sanderson S.C. (2015). Spatiotemporal evolution of Calophaca (Fabaceae) reveals multiple dispersals in central Asian mountains. PLoS ONE.

[8] Wu Z., Raven P., Hong D. (2010). Flora of China, Volume 10: Fabaceae.

[9] Zhang M., Xue J., Zhang Q., Sanderson S.C. (2015). Inferring ancestral distribution area and survival vegetation of Caragana (Fabaceae) in Tertiary. Plant Syst. Evol..

[10] Hou X., Liu J.E., Zhao Y.Z. (2008). Molecular phylogeny of Caragana (Fabaceae) in China. J. Syst. Evol..

[11] Zhang M., Fritsch P.W., Cruz B.C. (2009). Phylogeny of Caragana (Fabaceae) based on DNA sequence data from rbcL, trnS-trnG, and ITS. Mol. Phylogenet. Evol..

[12] Guo C., Liu K., Li E., Chen Y., He J., Li W., Dong W., Suo Z. (2023). Maternal donor and genetic variation of Lagerstroemia indica cultivars. Int. J. Mol. Sci..

[13] Li E., Liu K., Deng R., Gao Y., Liu X., Dong W., Zhang Z. (2023). Insights into the phylogeny and chloroplast genome evolution of Eriocaulon (Eriocaulaceae). BMC Plant Biol..

[14] Liu X., Bai Y., Wang Y., Chen Y., Dong W., Zhang Z. (2023). Complete Chloroplast Genome of Hypericum perforatum and Dynamic Evolution in Hypericum (Hypericaceae). Int. J. Mol. Sci..

[15] Dong W., Li E., Liu Y., Xu C., Wang Y., Liu K., Cui X., Sun J., Suo Z., Zhang Z. (2022). Phylogenomic approaches untangle early divergences and complex diversifications of the olive plant family. BMC Biol..

[16] Liu L., Li H., Li J., Li X., Hu N., Sun J., Zhou W. (2024). Chloroplast genomes of Caragana tibetica and Caragana turkestanica: Structures and comparative analysis. BMC Plant Biol..

[17] Jiang M., Chen H., He S., Wang L., Chen A.J., Liu C. (2018). Sequencing, Characterization, and Comparative Analyses of the Plastome of *Caragana rosea* var. rosea. Int. J. Mol. Sci..

[18] Yuan M., Yin X., Gao B., Gu R., Jiang G. (2022). The chloroplasts genomic analyses of four specific *Caragana* species. PLoS ONE.

[19] Olejniczak S.A., Lojewska E., Kowalczyk T., Sakowicz T. (2016). Chloroplasts: State of research and practical applications of plastome sequencing. Planta.

[20] Choi I.S., Jansen R., Ruhlman T. (2019). Lost and Found: Return of the Inverted Repeat in the Legume Clade Defined by Its Absence. Genome Biol. Evol..

[21] Jiao Y.X., He X.F., Song R., Wang X.M., Zhang H., Aili R., Chao Y.H., Shen Y.H., Yu L.X., Zhang T.J. (2023). Recent structural variations in the Medicago chloroplast genomes and their horizontal transfer into nuclear chromosomes. J. Syst. Evol..

[22] Lee C., Choi I.S., Cardoso D., de Lima H.C., de Queiroz L.P., Wojciechowski M.F., Jansen R.K., Ruhlman T.A. (2021). The chicken or the egg? Plastome evolution and an independent loss of the inverted repeat in papilionoid legumes. Plant J..

[23] Ping J., Hao J., Li J., Yang Y., Su Y., Wang T. (2022). Loss of the IR region in conifer plastomes: Changes in the selection pressure and substitution rate of protein-coding genes. Ecol. Evol..

[24] Jansen R.K., Wojciechowski M.F., Sanniyasi E., Lee S.B., Daniell H. (2008). Complete plastid genome sequence of the chickpea (Cicer arietinum) and the phylogenetic distribution of rps12 and clpP intron losses among legumes (Leguminosae). Mol. Phylogenet. Evol..

[25] Cai Z.Q., Guisinger M., Kim H.G., Ruck E., Blazier J.C., McMurtry V., Kuehl J.V., Boore J., Jansen R.K. (2008). Extensive Reorganization of the Plastid Genome of *Trifolium subterraneum* (Fabaceae) Is Associated with Numerous Repeated Sequences and Novel DNA Insertions. J. Mol. Evol..

[26] Sveinsson S., Cronk Q. (2014). Evolutionary origin of highly repetitive plastid genomes within the clover genus (Trifolium). BMC Evol. Biol..

[27] Jo I.-H., Han S., Shim D., Ryu H., Hyun T.K., Lee Y., Kim D., So Y.-S., Chung J.-W. (2022). Complete Chloroplast Genome of the Inverted Repeat-Lacking Species Vicia bungei and Development of Polymorphic Simple Sequence Repeat Markers. Front. Plant Sci..

[28] Millen R.S., Olmstead R.G., Adams K.L., Palmer J.D., Lao N.T., Heggie L., Kavanagh T.A., Hibberd J.M., Gray J.C., Morden C.W. (2001). Many parallel losses of *infA* from chloroplast DNA during angiosperm evolution with multiple independent transfers to the nucleus. Plant Cell.

[29] Jansen R.K., Saski C., Lee S.B., Hansen A.K., Daniell H. (2011). Complete plastid genome sequences of three Rosids (*Castanea*, *Prunus*, *Theobroma*): Evidence for at least two independent transfers of *rpl22* to the nucleus. Mol. Biol. Evol..

[30] Moghaddam M., Ohta A., Shimizu M., Terauchi R., Kazempour-Osaloo S. (2022). The complete chloroplast genome of Onobrychis gaubae (Fabaceae-Papilionoideae): Comparative analysis with related IR-lacking clade species. BMC Plant Biol..

[31] Weng M.-L., Blazier J.C., Govindu M., Jansen R.K. (2014). Reconstruction of the Ancestral Plastid Genome in Geraniaceae Reveals a Correlation between Genome Rearrangements, Repeats, and Nucleotide Substitution Rates. Mol. Biol. Evol..

[32] Liu H., Zhao W., Hua W., Liu J. (2022). A large-scale population based organelle pan-genomes construction and phylogeny analysis reveal the genetic diversity and the evolutionary origins of chloroplast and mitochondrion in *Brassica napus* L. BMC Genom..

[33] Xiong Y., Xiong Y.L., Jia X.J., Zhao J.M., Yan L.J., Sha L.N., Liu L., Yu Q.Q., Lei X., Bai S.Q. (2023). Divergence in *Elymus sibiricus* is related to geography and climate oscillation: A new look from pan-chloroplast genome data. J. Syst. Evol..

[34] Zhang J., Huang H., Qu C., Meng X., Meng F., Yao X., Wu J., Guo X., Han B., Xing S. (2021). Comprehensive analysis of chloroplast genome of *Albizia julibrissin* Durazz. (*Leguminosae* sp.). Planta.

[35] Yen L.-T., Kousar M., Park J. (2023). Comparative Analysis of Chloroplast Genome of Desmodium stryacifolium with Closely Related Legume Genome from the Phaseoloid Clade. Int. J. Mol. Sci..

[36] Ge S. (2022). A review of recent studies of plant systematics and evolution in China. Biodivers. Sci..

[37] Li J.L., Wang S., Yu J., Wang L., Zhou S.L. (2013). A Modified CTAB Protocol for Plant DNA Extraction. Chin. Bull. Bot..

[38] Bolger A.M., Lohse M., Usadel B. (2014). Trimmomatic: A flexible trimmer for Illumina sequence data. Bioinformatics.

[39] Jin J.-J., Yu W.-B., Yang J.-B., Song Y., dePamphilis C.W., Yi T.-S., Li D.-Z. (2020). GetOrganelle: A fast and versatile toolkit for accurate de novo assembly of organelle genomes. Genome Biol..

[40] Dong W., Liu Y., Li E., Xu C., Sun J., Li W., Zhou S., Zhang Z., Suo Z. (2022). Phylogenomics and biogeography of *Catalpa* (Bignoniaceae) reveal incomplete lineage sorting and three dispersal events. Mol. Phylogenet. Evol..

[41] Qu X.-J., Moore M.J., Li D.-Z., Yi T.-S. (2019). PGA: A software package for rapid, accurate, and flexible batch annotation of plastomes. Plant Methods.

[42] Beier S., Thiel T., Munch T., Scholz U., Mascher M. (2017). MISA-web: A web server for microsatellite prediction. Bioinformatics.

[43] Frazer K.A., Pachter L., Poliakov A., Rubin E.M., Dubchak I. (2004). VISTA: Computational tools for comparative genomics. Nucleic Acids Res..

[44] Suzuki R., Shimodaira H. (2006). Pvclust: An R package for assessing the uncertainty in hierarchical clustering. Bioinformatics.

[45] Chen C., Chen H., Zhang Y., Thomas H.R., Frank M.H., He Y., Xia R. (2020). TBtools: An Integrative Toolkit Developed for Interactive Analyses of Big Biological Data. Mol. Plant.

[46] Zhang D., Gao F., Jakovlic I., Zou H., Zhang J., Li W.X., Wang G.T. (2020). PhyloSuite: An integrated and scalable desktop platform for streamlined molecular sequence data management and evolutionary phylogenetics studies. Mol. Ecol. Resour..

[47] Xu B., Yang Z. (2013). PAMLX: A graphical user interface for PAML. Mol. Biol. Evol..

[48] Dong W., Xu C., Cheng T., Zhou S. (2013). Complete chloroplast genome of *Sedum sarmentosum* and chloroplast genome evolution in Saxifragales. PLoS ONE.

[49] Duan L., Li S.J., Su C., Sirichamorn Y., Han L.N., Ye W., Loc P.K., Wen J., Compton J.A., Schrire B. (2021). Phylogenomic framework of the IRLC legumes (Leguminosae subfamily Papilionoideae) and intercontinental biogeography of tribe Wisterieae. Mol. Phylogenet. Evol..

[50] Katoh K., Standley D.M. (2013). MAFFT multiple sequence alignment software version 7: Improvements in performance and usability. Mol. Biol. Evol..

[51] Capella-Gutiérrez S., Silla-Martínez J.M., Gabaldón T. (2009). trimAl: A tool for automated alignment trimming in large-scale phylogenetic analyses. Bioinformatics.

[52] Kalyaanamoorthy S., Minh B.Q., Wong T.K.F., von Haeseler A., Jermiin L.S. (2017). ModelFinder: Fast model selection for accurate phylogenetic estimates. Nat. Methods.

[53] Minh B.Q., Schmidt H.A., Chernomor O., Schrempf D., Woodhams M.D., von Haeseler A., Lanfear R. (2020). IQ-TREE 2: New models and efficient methods for phylogenetic inference in the genomic era. Mol. Biol. Evol..

[54] Ronquist F., Teslenko M., van der Mark P., Ayres D.L., Darling A., Hohna S., Larget B., Liu L., Suchard M.A., Huelsenbeck J.P. (2012). MrBayes 3.2: Efficient Bayesian phylogenetic inference and model choice across a large model space. Syst. Biol..

[55] Rambaut A., Drummond A.J., Xie D., Baele G., Suchard M.A. (2018). Posterior summarization in Bayesian phylogenetics using Tracer 1.7. Syst. Biol..

